# Striving for Triadic Collaboration in Pediatric Speech Sound Disorder Intervention: Grounded Theory Study

**DOI:** 10.2196/86364

**Published:** 2026-07-08

**Authors:** Soyoung Kyung, Youngsam Min, Byoung-a Min, Yuyoung Kim, Jinwoo Kim

**Affiliations:** 1 Human Computer Interaction Lab Graduate Program in Cognitive Science Yonsei University Seoul Republic of Korea; 2 Meta Design Lab Department of Industrial Design Konkuk University Seoul Republic of Korea; 3 HAII Corporation Seoul Republic of Korea; 4 Department of Business Administration Yonsei University Seoul Republic of Korea

**Keywords:** child participation, digital health, grounded theory, home-based practice, pediatric speech intervention, qualitative study, speech sound disorder, speech sound generalization, speech-language pathology, triadic collaboration

## Abstract

**Background:**

Collaboration between speech-language pathologists (SLPs) and parents is central to pediatric speech sound disorder (SSD) intervention. Prior research has emphasized the relational and home-practice components of collaboration; however, little is known about the enactment, organization, and maintenance of collaborative processes across SLP sessions and home practice in relation to speech sound generalization.

**Objective:**

This study examined the enactment and organization of collaboration between SLPs and parents in pediatric SSD intervention, with attention to children’s participation. Using a grounded theory approach, it aimed to develop an empirically grounded conceptual account of how collaboration is organized in relation to speech sound generalization.

**Methods:**

This qualitative study adopted a Strauss and Corbin–oriented grounded theory approach. Semistructured interviews were conducted with 12 SLPs and 10 parents of children aged 4 to 7 years receiving SSD intervention in South Korea. Data collection and analysis proceeded iteratively using open, axial, and selective coding with constant comparison. A paradigm model explicated relationships among categories, and selective coding integrated categories around a core category.

**Results:**

The central phenomenon was conceptualized as striving for triadic collaboration, reflecting SLPs’ and parents’ efforts to coordinate roles, expectations, and practice while supporting children’s participation across SLP sessions and home practice. Three interrelated action/interaction strategies were identified: SLP-led monitoring strategies, parent-led home-based intervention strategies, and gradual motivation strategies. These collaborative processes were shaped by background and practice-level constraints. A category integration diagram was developed to represent how the core category, action/interaction strategies, and contextual concerns were organized around triadic collaboration.

**Conclusions:**

The findings suggest that collaboration in pediatric SSD intervention is a contextually embedded process in which SLPs and parents coordinate roles and practices across SLP sessions and home practice while supporting children’s participation. The category integration diagram represents how collaborative processes were organized across settings in relation to the gap between structured target sound production and everyday speech use. These findings highlight the relevance of coordinated collaboration for supporting practice across settings in relation to speech sound generalization.

## Introduction

### Background

Speech sound disorders (SSDs) are among the most common communication disorders in early childhood, affecting approximately 8%-9% of preschool-aged children [1]. SSDs comprise a heterogeneous set of developmental difficulties in speech sound production, including phonological impairment, childhood apraxia of speech (CAS), childhood dysarthria, and articulation impairment, which differ in terms of underlying mechanisms and treatment approaches [2-5]. These challenges are described alongside phonological (linguistic) and motor speech (speech-motor planning, programming, or execution) dimensions [6,7]. Children with SSDs are at increased risk of language and literacy difficulties in later life [8,9]. Persistent speech errors are associated with social–emotional challenges and decreased participation within social and academic contexts [10].

In South Korea, SSDs comprise 44.1% of speech-language service caseloads, and children aged 4 to 7 years comprise approximately half of these cases [11]. Services are delivered in individual one-on-one sessions scheduled on a regular weekly basis, with children typically working with the same speech-language pathologist (SLP) across sessions to support treatment continuity. Parents are involved through brief consultations before or after therapy. These consultations last approximately 10 minutes and focus on reviewing session content and assigning home-based practice. Direct parent participation during therapy sessions is uncommon in many clinical settings, and parental involvement is therefore primarily structured through postsession consultations and home practice guidance. Coordinating therapy implementation within the home environment has become a crucial component of service delivery [12].

Clinical assessments use standardized tools that quantify speech accuracy and severity based on age-referenced norms [13]. Moreover, intervention approaches differ according to diagnostic subtype [5,14]. For example, contrast-based linguistic interventions address phonological impairments [14,15], targeting the reorganization of the phonological system [14,16]; meanwhile, CAS requires motor-based treatment targeting speech planning and sequencing [17,18], focused on accuracy and consistency of motor plans [17,19]. However, standardized assessment tools used in routine clinical settings often provide limited differentiation among SSD subtypes [20,21]. Consequently, in routine service contexts, children are broadly characterized by functional speech sound difficulties, and standardized subtype classification is not documented [22-24]. A key therapeutic objective is to promote generalization from trained stimuli to functional speech use across linguistic units and everyday communication contexts [25-27]. Achieving this generalization requires sufficient practice intensity [26,28].

Evidence suggests that high-intensity interventions—particularly those with increased session frequency and higher practice dose—may facilitate efficient gains associated with generalization [14,28]. However, sustaining high-intensity intervention in routine services is challenging owing to contextual constraints such as limited therapist availability, scheduling demands, and caregiver burden, contributing to the gap between evidence and practice [28-30]. Thus, extending practice opportunities beyond clinic-based intervention is important for supporting generalization [26,28].

SLPs collaborate with parents to extend clinic-based intervention into home-based contexts [31,32]. While relationship-based approaches consider family-specific constraints [31], several barriers hinder consistent home-based practice. Parents frequently face logistical demands, lack of readiness, and experience uncertainty regarding specific roles or activities [33]. Monitoring and supporting home-based practice across contexts are challenging for clinicians [34]. Consequently, clinical practice lacks a structured framework for defining participant roles, facilitating systematic interaction, and providing clear therapeutic guidance [35,36].

Scholars have discussed digital technologies in speech sound intervention as potential tools for alleviating spatiotemporal constraints and extending therapy continuity and practice intensity beyond the clinic into home-based contexts [37,38]. Prior research has mainly focused on their functional utility, including support for child engagement, intervention intensity, and associated outcomes [39-41]. However, research on digitally mediated collaboration among SLPs, parents, and children remains limited [41] and lacks a systematic examination of the organization and maintenance of digitally mediated collaboration under home constraints, including barriers to caregiver participation [42,43]. Studies on telepractice and remote service delivery have reported that digital technologies can enhance flexibility and accessibility and may expand opportunities for collaboration [43,44]. Challenges in rapport building and inequities in technology access may constrain participation [42,45]. These findings highlight the need to investigate the contribution of digitally mediated conditions to collaborative processes in pediatric speech sound intervention [41]. Prior research has highlighted the importance of collaboration between SLPs and parents in pediatric SSD intervention [31,32,46], which facilitates information exchange regarding children’s status while enabling the codevelopment of individualized treatment plans [31]. Speech sound objectives relevant to children’s daily life can be identified, and intervention strategies can be tailored [31,47]. Scholars have proposed realist-informed frameworks, such as preliminary program theory, for outlining the influence of contextual factors on collaborative practice [46].

Nevertheless, the SSD literature has frequently described collaboration in terms of discrete components, mechanisms, or facilitating and constraining conditions [31,32,46]. Existing studies offer limited guidance on the organization and maintenance of collaboration within clinical and home-based settings [31,46]. Qualitative studies have indicated that parents’ understanding of their roles and engagement in home-based practice may change during service engagement in response to family routines, competing demands, and ongoing interactions with services [33,36,48]. Few studies have examined the evolution of collaborative processes among SLPs, parents, and children over time. Moreover, research has paid limited attention to the implementation of collaborative processes and their role in sustained home-based practice and speech sound generalization [31,33,46]. Thus, a process-oriented examination is required to elucidate how collaboration is sustained across contexts.

### Research Objectives

This study aims to examine the enactment and organization of collaboration between SLPs and parents in everyday clinical settings for pediatric SSD intervention involving children with heterogeneous speech sound difficulties. It identifies the roles, interactions, and conditions that influence collaboration across SLP sessions and home practice. It examines children’s participation through SLP and parent narratives, focusing on how children are positioned within collaborative processes. Based on interview data on collaborative experiences, the study aims to develop an empirically grounded conceptual account of how collaboration is organized in relation to speech sound generalization.

## Methods

### Study Design

This qualitative study adopted a Strauss and Corbin–oriented grounded theory (GT) approach to examine real-world interactions among SLPs, parents, and children across clinical and home settings in pediatric speech sound intervention [49]. Analysis focused on interactional processes and contextual constraints through which collaboration was enacted across clinical and home-based contexts [31]. Semistructured interviews were conducted with 10 parents of children with SSDs and 12 SLPs. Children were not directly interviewed because of age and speech-language limitations [50]. The study examined children’s participation as described by parents and SLPs during interviews. Data collection and analysis proceeded iteratively using constant comparative methods. Analysis informed subsequent data collection and sampling, enabling the elaboration and refinement of emerging concepts [49]. Consistent with Strauss and Corbin’s GT, this study maintained a systematic analytic framework while enabling interpretive flexibility during analysis [49,51]. It used multiple strategies to enhance rigor, including analyst triangulation, member checking, and peer debriefing [51]. The first author, who conducted the interviews and led the analysis, is a PhD candidate in human–computer interaction with training in qualitative research. The research team included members with backgrounds in speech-language pathology and digital health. Analytic memos and team discussions were used to examine emerging interpretations. The reporting of this qualitative study was guided by the COREQ (Consolidated Criteria for Reporting Qualitative Research) checklist (Multimedia Appendix 1).

### Recruitment

The study used purposive sampling to recruit participants with various clinical experiences, service settings, and levels of involvement in pediatric speech sound intervention. SLPs and parents were independently recruited through social media platforms (eg, Instagram) and online communities. In the initial phase of recruitment, 10 SLPs and 8 parents from Seoul and surrounding metropolitan regions in South Korea were recruited.

Eligible parents were primary caregivers of children aged 4 to 7 years receiving speech-language therapy (SLT) for functional speech sound difficulties, including those formally diagnosed with SSD or clinically identified by certified SLPs. These children were currently undergoing or had completed professional SLT within the past 3 months. To focus on functional SSDs, cases involving identifiable organic or neurological conditions—including cleft palate, hearing impairment, intellectual disability, cerebral palsy, autism spectrum disorder, or chromosomal abnormalities—were excluded [2,3,52]. Most children were receiving face-to-face SLT at the time of the interviews, although 1 case involved remote service delivery. Diagnostic information was obtained from available clinical records provided by parents. Available documentation varied across cases; 5 cases included percentage of consonants correct indicators. Additionally, as supplementary information on functional speech intelligibility, Intelligibility in Context Scale scores were collected for all children (range: 2.29-4.29). Standardized subtype classification (eg, phonological vs motor-based subtypes), standard scores for speech production tests, and oral motor assessment results were not routinely documented in clinical records. Information about specific treatment approaches was not documented in the available clinical records. Eligible SLPs were required to have treated at least 5 children with SSDs. All participants were informed of the study objectives and procedures and provided written informed consent electronically via a secure online platform.

Data collection and analysis proceeded concurrently [49]. Guided by emerging analytic categories, the second phase of the recruitment was conducted using theoretical sampling [49,51]. Two SLP–parent dyads (2 SLPs and 2 parents) were recruited through snowball sampling. The members of both dyads were engaged in ongoing therapy with the same child, thus enabling examination of the alignment and negotiation of collaborative expectations within shared therapeutic contexts. The SLPs recommended parents who were eligible to participate.

### Data Collection

Data were collected between June and August 2024 using semistructured, in-depth interviews. Interview guides were developed based on the study aims and refined through pilot testing. They were further tailored using prequestionnaire responses and revised in response to ongoing analysis, consistent with the GT methodology [53]. Early interviews broadly explored collaborative practices, while later interviews incorporated focused questions to elaborate and refine emerging themes [49,53]. A brief preonline questionnaire collected contextual information, including parental level of education, session frequency, presence of home practice assignments, and perceived importance of home practice.

Individual interviews with SLPs and parents were conducted by the first author and lasted approximately 60 to 90 minutes. Interviews were conducted in a one-on-one format to support confidentiality and minimize response biases. No prior relationships existed between the interviewer and participants. Interviews were held at locations preferred by the participants, including homes, therapy rooms, and meeting spaces. Participants were informed that the study focused on collaborative practices in pediatric SLT. They were asked to describe their children’s participation and interactions during therapy and home-based practice.

All interviews were audio-recorded and transcribed using an automatic speech recognition tool (Clova Note; NAVER Corporation), followed by manual verification. The first author took field notes to capture contextual details and nonverbal cues. Analytic memos were taken during and after the interviews to document emerging themes and inform subsequent data collection and analysis [49]. Videos and photographs were shown during interviews for contextual illustration only, but were neither collected nor analyzed due to child privacy considerations.

Follow-up interviews were conducted via videoconferencing (Zoom; Zoom Communications Inc) or email when clarification was required during analysis. Data collection continued until theoretical saturation was reached, defined as the point at which additional data no longer contributed to the elaboration of categories and their relationships [51]. Multimedia Appendix 2 outlines the initial interview protocols, originally developed in Korean and translated into English summary tables for reporting.

### Data Analysis

Data analysis followed Strauss and Corbin’s GT approach using open, axial, and selective coding with constant comparison [49]. Data analysis and collection were conducted simultaneously, enabling emerging concepts to inform analytic focus [49,53].

During open coding, interview transcripts and field notes were examined per line to identify actions, interactions, and conditions [49]. Analytic memos documented emerging concepts and supported comparison across interviews. To calibrate analytic focus and coding boundaries, 3 researchers independently coded 2 interview transcripts (1 with a parent and 1 with an SLP) and engaged in analytic discussions. This process resulted in an initial set of open codes and provisional categories (Multimedia Appendix 3). The analytic coding framework was used as a working tool and iteratively revised as categories were compared, integrated, and elaborated through team discussion.

Axial coding focused on relating categories to subcategories by examining relationships among conditions, action/interaction strategies, and consequences. A paradigm model was used as an analytic tool for organizing these relationships [49,51]. Comparisons across participant groups (SLPs and parents) and contexts (clinical and home-based settings) supported refinement of the properties and dimensions of the categories.

Selective coding included the integration of categories around a core category representing the central collaborative process [49]. Categories were integrated to examine coherence and explanatory adequacy. Data from SLP–parent dyads were used to examine whether these relationships were sustained across shared therapeutic experiences, further refining the core category.

Children’s participation was analyzed through SLP and parent narratives, focusing on adults’ descriptions of guiding children in participation and the enactment of children’s behaviors and expressions during adult–child interactions. Atlas.ti (ATLAS.ti Scientific Software Development GmbH) supported initial coding and management of coded excerpts. Excel (Microsoft Corporation) was used to document the evolving coding framework and track analytic decisions during team discussions.

### Ethical Considerations

This study received ethical approval from the Yonsei University Institutional Review Board (number 7001988-202406 -HR-2290-03). Participants provided informed consent and were assured of their right to withdraw at any time without penalty. Data were anonymized and stored securely on an encrypted server. The participants received monetary compensation for their participation.

## Results

### Participant Characteristics

The study recruited 22 participants (10 parents of children with SSDs and 12 SLPs). Among the parents, 7 (70%) and 3 (30%) were aged in their 30s and 40s, respectively, while 6 (60%) and 4 (40%) resided in Seoul and nearby metropolitan areas, respectively. Regarding education, 1 (10%), 6 (60%), and 3 (30%) completed high school, a bachelor’s degree, and a master’s degree, respectively.

The children were evenly split by gender (boys: 5/10, 50%; girls: 5/10, 50%) and aged 4 to 6 years, with 5 (50%), 2 (20%), and 3 (30%) aged 4, 5, and 6 years, respectively. Intelligibility in Context Scale scores (5-point parent-rated scale) ranged from 2.29 to 4.29. Among 5 cases with available percentage of consonants correct data, values ranged from 70.83% to 90.69%. Clinical records lacked diagnostic subtype classification; therefore, cases were described as functional speech sound difficulties. Service settings included hospital-based centers, private clinics, and 1 case of home-based therapy. The duration of therapy was less than 1 year for 3 (30%) children, 1-2 years for 4 (40%), and more than 2 years for 3 (30%). Eight children were actively receiving intervention, while 2 had completed intervention within the past 3 months. All children received SLT once or twice per week. Eight parents reported receiving home assignments; 2 reported none. Most parents reported inconsistent home practice because of time constraints; 1 reported consistent implementation. All parents perceived home practice as influencing therapy outcomes, while 3 described it as crucial. Information on specific therapy methods was obtained during parent interviews, as it was unavailable in clinical records.

Among the SLPs, 8 (67%) were aged in their 20s, 2 (17%) were in their 30s, and 2 (17%) were in their 40s. The majority worked in private clinics (7/12, 58%) or hospital-based centers (5/12, 42%), held a master’s degree (7/12, 58%) or were enrolled in graduate programs (2/12, 17%) and had clinical experience of less than 4 years (4/12, 33%), 4-8 years (5/12, 42%), and more than 8 years (3/12, 25%). Regarding SSD caseloads, 6 (50%), 4 (33%), and 2 (17%) treated more than 20, 10-20, and fewer than 10 children, respectively. Multimedia Appendix 4 provides detailed participant characteristics.

### Central Phenomenon: Striving for Triadic Collaboration

Through interviews, collaboration between SLPs and parents was described as an ongoing effort to align roles, expectations, and practices within clinical and home-based settings. Participants emphasized that collaboration required ongoing adjustment rather than agreement: “It’s not that we just follow instructions. We try, and then we adjust depending on how things actually work at home” (P04).

Collaboration was enacted not as passive adherence but as a process of repeated recalibration. Accordingly, the central phenomenon was conceptualized as “striving for triadic collaboration.”

Striving unfolded within a clinical context characterized by fragmented consultation times, cultural reliance on professional expertise, and digital ambivalence. Digitally mediated conditions supported cross-context communication and progress monitoring but raised concerns regarding diminished face-to-face engagement and technological overreliance. Expectations regarding roles and responsibilities centered on the SLP. Several causal conditions intensified this striving. Limitations in face-to-face therapy, interactional discrepancies, and challenges in achieving speech sound generalization were described as influencing efforts to extend practice beyond clinic sessions.

Participants described efforts to bridge the clinic–home gap as the formation of cooperative partnerships. This process involved elucidating roles, negotiating expectations, and extending practice beyond therapy sessions. SLPs adjusted clinical guidance to align with family routines and children’s responses, while parents engaged in repetitive attempts to implement and refine home-based practice: “Even when we agree on the plan, it doesn’t always unfold the way we expected” (SLP05).

Collaboration unfolded not as a fixed agreement but as an ongoing process requiring recalibration in response to contextual constraints, children’s responses, and changes in parental attitudes. Through these adjustments, collaboration evolved over time.

**Figure 1:**
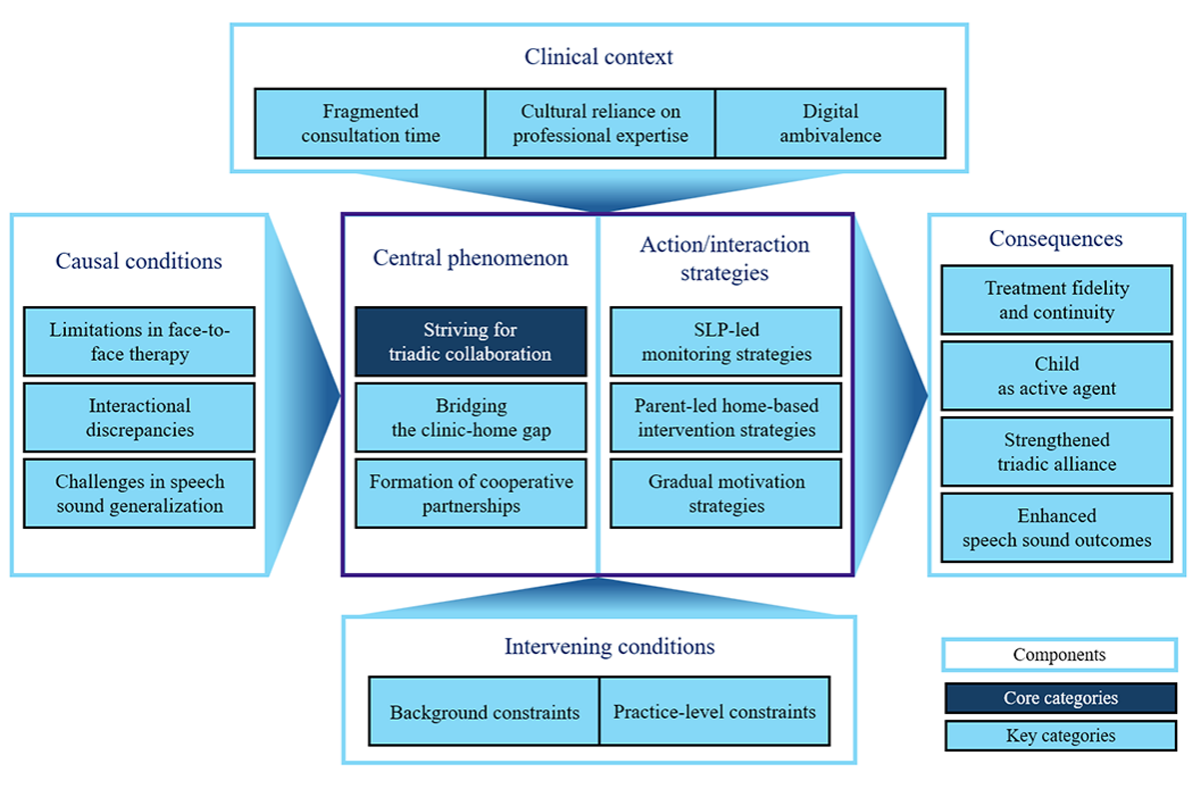
Paradigm model of striving for triadic collaboration. Derived through axial coding, this model illustrates relationships among causal conditions, intervening conditions, the central phenomenon, action/interaction strategies, and consequences. SLP: speech-language pathologist.

Within the paradigm model, striving for triadic collaboration served as a central phenomenon linking causal conditions, action/interaction strategies, and consequences. Clinical contexts and causal conditions influenced this striving, which was enacted through the action/interaction strategies presented in the following section. These processes were reflected in treatment fidelity and continuity, children’s participation as active agents, a stronger triadic alliance, and changes observed in speech sound performance. Figure 1 presents the paradigm model illustrating these relationships. Multimedia Appendix 5 provides a detailed coding matrix outlining relationships among components, key categories, and subcategories within the paradigm model.

### Action/Interaction Strategies for Triadic Collaboration

#### Overview

A total of 3 interrelated action/interaction strategies were identified in response to the central phenomenon of striving for triadic collaboration: SLP-led monitoring strategies, parent-led home-based intervention strategies, and gradual motivation strategies. These strategies were enacted in response to clinical and causal conditions and addressed the clinic–home gap. SLP-led monitoring strategies facilitated goal alignment and progress tracking; parent-led home-based intervention strategies extended therapeutic practice into daily contexts; and gradual motivation strategies supported children’s engagement in therapeutic tasks. Table 1 summarizes the paradigm-informed coding structure underlying these action/interaction strategies, including key categories and subcategories derived through open and axial coding.

**Table 1:** Key categories, subcategories, and key codes associated with the action/interaction strategies identified within the paradigm model.

Key categories and subcategories			Key codes
SLP^a^-led monitoring strategies			
	Collaborative goal setting	Explain children’s condition objectively to parents Set therapy goals that reflect children’s status and parents’ needs Establish stepwise goals based on difficulty levels	
	Transparent communication	SLPs share therapy progress and updates Parents share observations in daily life Collaborate with preschool and kindergarten teachers	
	Progress monitoring	Visualize progress using graphs and metrics Compare pretherapy and posttherapy outcomes Adjust detailed goals according to status changes	
Parent-led home-based intervention strategies			
	Parent coaching supported by SLP	Provide home practice guidelines Encourage and positively reinforce parents’ efforts	
	Parent-led speech practice	Facilitate consistent child practice Manage children’s stress during home practice	
	Guided self-monitoring	Provide visual and auditory discrimination tasks Offer appropriate cues during practice Share feedback to promote accurate speech production Create opportunities for self-correction	
	Contextualized practice	Promote generalization through repeated practice in diverse linguistic contexts Simulate peer interaction scenarios Encourage generalization in various locations with family support	
Gradual motivation strategies			
	Choice-supportive interaction	Incorporate preferred activities into therapy (eg, toys, games, and play) Apply gamification elements (eg, collection, missions, and challenges) Offer structured choices during activities (eg, selecting tasks or materials)	
	Incentive-based participation	Provide tangible rewards (eg, snacks and drinks) Use activity-based rewards (eg, playtime and games) Visualize effort and progress (eg, stickers, stamps, and tracking charts)	
	Socially mediated reinforcement	Deliver verbal reinforcement (eg, praise and encouragement) Use nonverbal reinforcements (eg, high-five and clapping) Facilitate social recognition (eg, sharing achievements with family members)	

^a^SLP: speech-language pathologist.

#### SLP-Led Monitoring Strategies

##### Overview

SLPs described monitoring as a strategy for maintaining collaboration with parents and tracking children’s progress. Monitoring involved 3 interconnected elements: collaborative goal setting, transparent communication, and progress monitoring.

##### Collaborative Goal Setting

Collaborative goal setting was described as a process in which SLPs and parents developed a shared understanding of children’s status and negotiated therapy goals. SLPs discussed therapy goals with parents based on formal and informal assessment results and positioned this discussion as an initial step in collaborative work.

If the mother shares her goals, I try to incorporate them. If they seem appropriate, I include them; if it is too high, I explain why. I set goals together with the mother.

SLP02

In clinical settings, parents were often reluctant to express their opinions, citing respect for the expertise of the SLP. This pattern was associated with challenges in collaborative goal setting.

The moment a mother says something, she might think she’s expressing dissatisfaction with the teacher’s curriculum.

P09

Parents had limited access to information enabling an objective understanding of their child’s status. They reported difficulty in assessing the necessity of therapy and the appropriateness of goals without standardized assessment results.

We did an assessment, but I didn’t receive a result sheet. They didn’t give it to me. So, I had to listen and take notes...because I need to know too.

P02

Parents expressed the need for assessment results in an understandable format.

It would be good if we could see something like that. Within the children’s average range... (so we can know our child’s position) similar to pediatric health checkups...

P01

Receiving assessment results in a format similar to that used in pediatric health checkups was described as supporting parental understanding.

##### Transparent Communication

Participants described mutual information exchange about children’s progress as part of maintaining collaborative relationships. SLPs sought updates on children’s daily lives, while parents expected feedback from therapy sessions. However, time constraints due to back-to-back therapy schedules frequently limited these exchanges.

During consultations, I feel extremely rushed. The consultation ends at the same time the next child’s class begins...There’s so much to talk about, but time is really tight.

SLP12

Parents also recognized these constraints and expressed regret about insufficient communication. Consultation time was approximately 5 minutes per session.

In 5 minutes, the consultation time is extremely short for asking questions and getting answers.

P09

In response to these limitations, SLPs attempted asynchronous communication using digitally mediated communication tools. This approach, however, depended on parental participation.

I asked them to record videos of the child practicing. But the mother did it for a while but later asked to stop because it was very demanding.

SLP06

These structural and practical barriers limited consistent communication and information exchange between home-based practice and clinic-based intervention.

##### Progress Monitoring

Progress monitoring was described as a part of maintaining collaboration. SLPs and parents reported that presenting comparative therapy data influenced parental perceptions of therapy.

What parents are most curious about is knowing the numbers—how well the child is doing right now.

SLP05

SLPs described changes in parental perceptions when objective comparative data were provided. One SLP described playing before-and-after recordings to a parent.

The mother didn’t really know how unclear her child’s pronunciation was. I sent her part of a recording from class...after that she said, “When I listened to just the voice without any context, I could really feel that my child had improved.”

SLP08

Parents similarly reported that listening to recordings supported clearer recognition of their child’s speech difficulty and therapy progress.

I told the teacher that I didn’t think my child’s pronunciation was that bad. But after listening to the recording, I could really hear the difference and realized the improvement.

P07

Some parents referenced familiar technology platforms, such as Wink, when discussing potential monitoring formats.

Like Wink (a customized learning device for young children), it would be good if teachers could share the child’s activities and data that parents can see.

P09

Participants described objective digital measurement tools as facilitating shared access to therapy-related information.

#### Parent-Led Home-Based Intervention Strategies

##### Overview

SLPs described continued speech practice at home as part of extending therapy beyond clinic sessions. Parent-led home-based intervention involved 4 interconnected elements: parent coaching supported by SLP, parent-led speech practice, guided self-monitoring, and contextualized practice.

##### Parent Coaching Supported by SLP

SLPs described systematic coaching as part of supporting parent-led home practice, as many parents lacked familiarity with specific guidance methods. Parents demonstrated varying levels of awareness and ability to implement home practice, and many reported difficulty in maintaining it over time. SLPs recognized this gap and provided specific methods to connect therapy-room activities with home-based practice.

I let them (parents) feel how my mouth moves, and I also help position theirs, such that they understand the articulation; however, with their daily routines and work, they often forget this technique.

SLP10

In response to these limitations, SLPs developed strategies for providing recorded materials with explanations.

When I edit and bring recorded materials, we listen to them together and I explain, “This is how I provided stimulation, and at this point the child produced correct articulation.”

SLP12

Parents expressed anxiety about providing feedback as nonexperts, which was associated with more passive responses: “If I do this wrong, I might actually create bad habits” (P06).

When I really can’t understand the pronunciation, I just say “good” and move on. I don’t keep asking about it.

P08

Three parents enrolled in related academic programs or obtained certifications to provide therapy more formally. This process required considerable time and introduced additional difficulties.

I started grad school hoping to help my child more with his therapy but juggling school, practicum hours, and everything at home frequently meant I had to leave him with a sitter, and I ended up being less engaged in his day-to-day therapy.

P03

This case reflected challenges in sustaining home practice through individual parental effort and highlighted the need for ongoing professional support.

##### Parent-Led Speech Practice

Participants described challenges in sustaining speech sound practice at home. Parents reported difficulty in ensuring sufficient practice time due to constraints such as work demands, caring for siblings, and household tasks.

I think there are definitely areas that improve if you consistently practice at home too. But it’s hard to practice at home. It’s difficult to get them to sit down, and I’m tired too.

P02

Lack of child cooperation added to challenges in home practice: “They don’t want to do homework, and they don’t sit for long periods, so sometimes we can’t do it” (P04).

Parents limited practice to avoid stressing the children, whereas SLPs emphasized the value of continued home practice.

My child said, “Do you think here is a lip teacher’s academy?” (during COVID-19, therapists wore clear masks that only showed their mouths). So, I thought I should not stress them anymore, and I don’t force them to do this at home.

P02

SLPs expressed understanding of the parents’ practical difficulties while expressing concern about reduced home practice.

I understand being busy, but it’s a bit disappointing. It seems like a child could maintain progress if they practiced a little, so it’s unfortunate.

SLP09

Across interviews, SLPs emphasized the value of home practice, whereas parents described implementation challenges. Divergent perspectives between SLPs and parents emerged consistently.

##### Guided Self-Monitoring

Guided self-monitoring strategies focused on supporting children’s ability to monitor and correct their speech sounds. SLPs described self-monitoring as related to speech sound generalization.

The ultimate goal for these children is to monitor themselves without anyone else’s help and think “I just made a mistake, I need to do it again”—that’s the goal of all therapy.

SLP07

Given this purpose, SLPs provided cues that encouraged children to recognize errors before providing correction.

Let them think and try as much as possible on their own, and only if that doesn’t work, provide the correct sound. The priority is not to give them the answer right away, but to let them figure it out first.

SLP01

SLPs described self-evaluation using mobile phone video or recording functions as an effective strategy.

If “tiger (Horangi (호랑이))” inconsistently becomes “horanee” or “horiri”...I show children their own appearance and let them hear their own sounds. The process of making them aware is necessary.

SLP11

Parents noted that digital recording functions were useful for supporting self-monitoring during home-based practice and mentioned specific tool functionalities.

The recording button should be available for repetitive listening...so they can press it many times enough to catch that something is strange by listening to it.

P09

SLPs and parents described tools that increased awareness of speech errors and supported children’s self-monitoring behaviors (error recognition and self-correction) at home.

##### Contextualized Practice

Participants described contextualized practice as a way to support children’s use of target sounds beyond structured SLP sessions. SLPs emphasized that accurate production during brief clinical sessions did not necessarily extend to everyday speech use without continued home practice.

Generalization is practically impossible if we rely only on one 40-minute session a week. The mother needs to guide this at home.

SLP02

Because meeting once or twice a week is too short, generalization does not really happen. If the child does well only here and then leaves, it goes back to the same.

SLP06

SLPs described everyday communication contexts as important because children needed to use target sounds with different people and in comfortable settings, not only during structured interaction with the therapist.

To help a child speak well with any person and in any place—not only speaking well because they are tense in front of the therapist—I think support through home practice is needed.

SLP10

Parents described successful peer communication as a primary goal of therapy, noting that their children frequently struggled to interact with friends.

A frequently asked question was “What?”, while many kids commented, “I don't understand what you’re saying at all”...They got pointed out a lot by other kids...Occasionally, they didn’t interact and couldn’t get along with other children.

P05

SLPs and parents described peer interaction, story materials, play, and daily routines as contexts for practicing target sounds in varied interactions. SLPs also described gradual increases in practice complexity as relevant to children’s use of target sounds in spontaneous speech.

If we practice at the word, phrase, and sentence levels first, then when the child says what they really want to say to someone in a longer unit, they can develop control. I don’t think that ability suddenly appears when we move to sentences or conversation without practice at the word level.

SLP12

They don’t like it when it feels too formal or like studying, so we end up playing a lot of word chain games—whether we’re driving somewhere or just going about our day.

P01

These accounts described contextualized practice as connecting structured target sound production in SLP sessions with home routines and everyday speech use.

#### Gradual Motivation Strategies

##### Overview

SLPs and parents reported that maintaining children’s motivation was important during therapy, as children’s willingness to participate shaped therapy activities. SLPs adjusted motivational strategies according to children’s developmental level and therapy progression. Three subcategories were identified within gradual motivation strategies: choice-supportive interaction, incentive-based participation, and socially mediated reinforcement.

##### Choice-Supportive Interaction

SLPs used children’s interests and preferences as starting points to foster enjoyment and sustain participation in therapy. They identified these interests during initial consultations and integrated them into sessions by creating customized materials or introducing play-based elements.

I try to proceed according to children’s interests as much as possible. Characters the child likes or games they enjoy—I blend these in more—I make personalized materials directly.

SLP12

SLPs also described offering structured choices, such as allowing children to select materials or activity order, as a way to support participation during repetitive speech practice. These choice-supportive strategies were described as helping children engage with therapy tasks while maintaining a sense of involvement in the activity.

##### Incentive-Based Participation

SLPs and parents described using external reinforcement strategies to support children’s participation during repetitive practice. These strategies included tangible rewards, sticker charts, and activity-based rewards. SLPs described shifting from material rewards to activity-based rewards as therapy progressed.

Since we have 40-minute classes, if they concentrate for 30 minutes and finish all these tasks, then they can choose and do activities, board games, toys, or whatever they want for the remaining 10 minutes!

SLP12

Parents also used rewards during home practice, but described difficulty adjusting the timing and frequency of reinforcement.

At first, I gave them a gift every time they did well, but they started expecting something for even the smallest things. The therapist said the rewards were too frequent, so I switched to giving a gift only after they filled up their grape chart (sticker chart).

P07

These accounts described incentive-based participation as a way to support children’s cooperation while adjusting reward type and frequency across SLP sessions and home practice.

##### Socially Mediated Reinforcement

SLPs and parents described socially mediated reinforcement as a way to sustain children’s participation through praise, encouragement, and recognition from others. SLPs emphasized process-oriented praise that recognized children’s effort during difficult speech tasks rather than simple result-oriented praise.

I tell children, “Producing the right sounds is really, really hard—even for older kids—but just trying to practice is already amazing. You’re doing such a great job.”

SLP12

Parents described learning these specific praise methods from SLPs and applying them at home. They further extended social reinforcement by sharing children’s achievements with family members to provide social recognition.

I say, “Let’s send a video to grandma and grandpa too,” then grandma and grandpa call and say “you did so great,” and when this continues, the child wants to practice more.

P05

These accounts described socially mediated reinforcement as a way to support children’s participation through praise, encouragement, and family recognition across SLP sessions and home practice.

### Intervening Conditions

#### Overview

Intervening conditions described by parents and SLPs shaped how the 3 action/interaction strategies were enacted. These conditions were grouped into background constraints and practice-level constraints. Background constraints reflected participants’ caregiving responsibilities, work demands, emotional burden, and scheduling constraints, whereas practice-level constraints reflected gaps in feedback continuity, limited visibility of home practice and progress, and burdens associated with material and documentation management across SLP sessions and home practice.

#### Background Constraints

Parents and SLPs described workload and burnout as limiting sustained engagement in therapy-related activities. Parents reported difficulty maintaining home practice while managing caregiving responsibilities, sibling schedules, and work demands. One parent described being unable to give sufficient attention to the child receiving therapy because she also had to support an older child’s schoolwork and was too exhausted after work to complete assigned practice activities (P04). SLPs also described workload constraints in clinical settings. One SLP reported seeing 9 to 10 children consecutively in a day, leaving little time for breaks or extended postsession consultation (SLP12).

Participants also described emotional burden as a background constraint. Parents reported anxiety, guilt, and distress related to their child’s developmental trajectory, including concerns about delayed speech, possible disability, and whether they had sought services early enough (P07). SLPs described emotional labor when working with children who resisted therapy tasks or when communicating difficult feedback to parents. One SLP reported that repeated negative feedback and the need to manage children’s strong emotional responses sometimes required suppressing her own emotions during sessions (SLP03).

Participants further described scheduling constraints as limiting the regularity and intensity of intervention. Parents coordinated SLT with sibling care, schoolwork, private education, and other rehabilitation services. One parent described moving between an older child’s schedule and the younger child’s center-based therapy (P09). SLPs similarly noted that some children were managing multiple activities and center visits, making it difficult for families to add or maintain twice-weekly SLT sessions (SLP10). These constraints were described as opportunities for consistent parent-led practice and for intervention at the frequency SLPs sometimes recommended.

#### Practice-Level Constraints

Parents and SLPs described practice-level constraints involving gaps in feedback continuity, limited visibility of home practice and progress, and burdens associated with material and documentation management across SLP sessions and home practice.

First, participants described gaps in feedback continuity between SLP sessions and home practice. Communication often depended on brief postsession consultations, which limited opportunities for detailed discussion of children’s progress, home practice difficulties, or changes in family routines. Some parents described receiving only brief updates when sessions were scheduled back-to-back or when consultation time was embedded within a short therapy slot (P09). One parent explained that, in a hospital-based setting, she had to interrupt the final minutes of the child’s session to ask questions, which made consultation difficult because she also wanted the child to use the full therapy time (P03). SLPs similarly described consultation as compressed by clinical schedules, with limited time to explain what had occurred during the session and how practice should continue at home (SLP12). Thus, feedback was present but often brief or fragmented.

Second, participants described limited visibility of home practice and progress. Parents reported difficulty judging changes in their child’s speech because they had become accustomed to the child’s pronunciation patterns in daily life. In some cases, parents recognized progress only after comparing recordings or hearing feedback from another person (P07). SLPs also described uncertainty about whether children used target sounds outside the therapy room because they could not directly observe home or everyday communication contexts (SLP01). This constraint was especially relevant when children produced target sounds during structured SLP sessions but did not consistently use them in spontaneous speech or home routines.

Third, participants described material and documentation management as a practice-level burden. SLPs prepared paper-based worksheets, individualized word lists, and activity materials for each child, while parents were expected to store, retrieve, and use these materials during home practice. Parents described difficulties when paper worksheets were misplaced, forgotten, or difficult to organize across repeated sessions (P10). SLPs also described the burden of creating, modifying, and checking practice materials. One SLP described using Google Forms and generative artificial intelligence to prepare vocabulary quizzes, but still needed to manually check and revise materials before use (SLP05). These accounts indicated that material and documentation management were part of the work required to connect SLP sessions with home practice.

Together, these practice-level constraints described limitations in how SLP sessions and home practice were connected. Brief consultation time, limited access to children’s performance outside the clinic, and burdens associated with material and documentation management were described even when parents and SLPs were engaged in supporting children’s speech practice.

### Integration of Categories Around the Core Category

**Figure 2:**
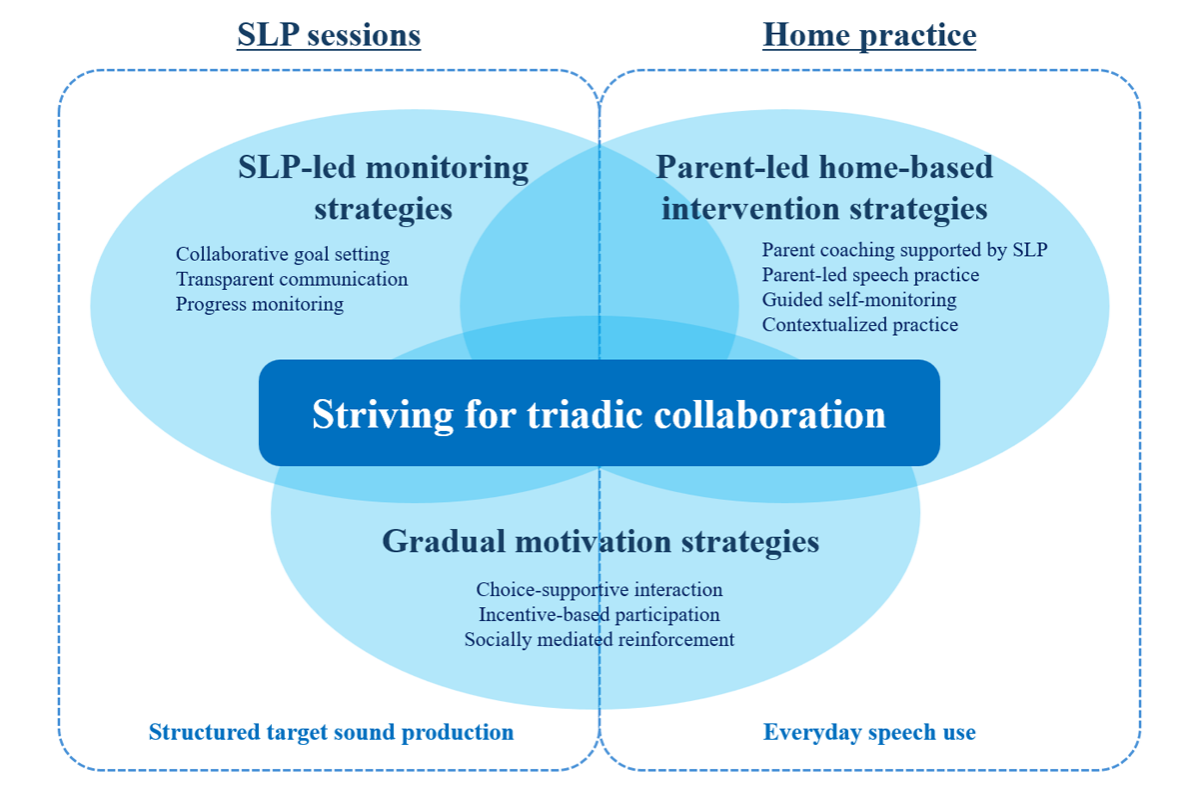
Category integration diagram of striving for triadic collaboration. The diagram illustrates how speech-language pathologist (SLP)–led monitoring strategies, parent-led home-based intervention strategies, and gradual motivation strategies were organized as overlapping components around the core category of striving for triadic collaboration across SLP sessions and home practice. Structured target sound production and everyday speech use are shown as contextual concerns described by participants.

During selective coding, the identified categories were integrated around the core category of striving for triadic collaboration. This core category captured participants’ accounts of collaboration as an ongoing effort to coordinate roles, expectations, and practice across SLP sessions and home practice. As described in the contextualized practice category, participants’ accounts repeatedly pointed to a gap between structured target sound production in SLP sessions and children’s everyday speech use. Structured target sound production and everyday speech use were included in Figure 2 as contextual concerns through which participants described the clinic–home gap.

Within this integration, SLP-led monitoring strategies, parent-led home-based intervention strategies, and gradual motivation strategies were organized as overlapping components rather than separate steps. Their overlap reflected participants’ accounts that the clinic–home gap required coordination among clinical guidance, home-based practice opportunities, and children’s engagement. SLP-led monitoring strategies supported shared understandings of therapy goals, children’s progress, and expectations for home practice. Parent-led home-based intervention strategies described how practice guidance was taken up, adapted, or constrained within family routines. Gradual motivation strategies operated across both settings because children’s participation had to be supported during structured SLP sessions as well as repeated home practice.

Taken together, these categories represented collaboration as a contextually embedded process of continued adjustment among SLPs, parents, and children. Figure 2 provides a visual representation of category integration derived through selective coding. The dashed areas indicate the 2 settings in which participants described collaborative work: SLP sessions and home practice. The overlapping ovals represent the 3 action/interaction strategies—SLP-led monitoring strategies, parent-led home-based intervention strategies, and gradual motivation strategies—and their subcategories as described above. The central bar represents the core category of striving for triadic collaboration, around which these strategies were integrated. Structured target sound production and everyday speech use are shown as contextual concerns that reflected participants’ descriptions of the gap between structured SLP sessions and daily speech use.

## Discussion

### Principal Findings

The findings provide an empirically grounded account of how SLPs and parents organized collaboration across SLP sessions and home practice in relation to the gap between structured target sound production and children’s everyday speech use. The core category, striving for triadic collaboration, captured collaboration as an ongoing effort by SLPs and parents to coordinate roles, expectations, and practice while supporting children’s participation. This collaboration was enacted through 3 action/interaction strategies: SLP-led monitoring strategies, parent-led home-based intervention strategies, and gradual motivation strategies. These strategies were shaped by background and practice-level constraints, including workload and burnout, emotional burden, scheduling constraints, gaps in feedback continuity, limited visibility of home practice and progress, and material and documentation management burden. Together, the findings suggest that collaboration in pediatric SSD intervention was organized as a contextually embedded process of continued adjustment across SLP sessions and home practice.

### Triadic Collaboration as an Organizing Process Across Contexts

Prior research has described collaboration in pediatric SLT as involving relational practices and joint planning between SLPs and parents [31,46,54]. Shared decision-making has also been identified as a key component of pediatric care [55]. Family-centered practice further emphasizes negotiated partnership, mutual understanding, and shared responsibility in service planning [56,57]. Recent work on collaborative relationships between families and SLPs also highlights communication, trust, and shared responsibility within early intervention contexts [58].

The present findings build on this literature by conceptualizing collaboration as a triadic organizing process involving SLPs, parents, and children. This formulation is consistent with research on triadic pediatric consultations, which suggests that children’s participation can reshape interactional dynamics rather than positioning children as passive recipients of care [59]. In this study, striving for triadic collaboration captured how participants described the coordination of roles and practices across SLP sessions and home practice.

In interpreting these findings, the 3 action/interaction strategies may be understood as a tentative, nonsequential conceptual lens of alignment, expansion, and internalization. SLP-led monitoring may support alignment by helping SLPs and parents share information about goals, progress, and expectations for home practice. Parent-led home-based intervention may expand practice opportunities into family routines. Gradual motivation strategies may support internalization by helping children move from externally prompted participation toward more self-directed engagement in repeated practice across SLP sessions and home routines.

### Collaboration in Relation to Speech Sound Generalization

In relation to speech sound generalization, the findings suggest that collaboration may be relevant because it organizes how practice, feedback, and child participation are coordinated across SLP sessions, home routines, and everyday speech use. Prior SSD research has identified self-monitoring as relevant to generalization [60]. Motivation and engagement have also been discussed in relation to sustained participation in learning and therapeutic contexts [61,62]. Motor learning and speech practice research emphasizes practice conditions, repetition, and feedback in speech intervention [63,64].

Evidence on parent involvement in childhood SSD intervention emphasizes extending practice beyond therapy sessions and supporting home practice [31-33]. Studies of parental roles and coworking further show that home practice is shaped by parents’ beliefs, role expectations, and family routines [36,48].

The present findings connect these strands by situating these practices within collaborative work across SLP sessions and home practice. Guided self-monitoring was described as one component of parent-led home-based intervention. Contextualized practice was described as connecting structured target sound production with home routines and everyday communication contexts. These SLP- and parent-supported practices were described as creating opportunities for children to notice and adjust speech production during practice.

Broader work on self-regulation and motivation further supports attention to how children participate in learning and therapeutic activities [65,66]. In this framing, guided self-monitoring is best understood as a participant-described practice within collaborative intervention, with its relation to speech sound generalization requiring further study. Future studies could examine whether and how adult-supported collaborative practices contribute to children’s independent self-monitoring and speech sound generalization over time.

The organization of triadic collaboration may also vary according to children’s clinical presentation, treatment focus, family resources, and service context. SSD includes heterogeneous clinical presentations, and diagnostic and dimensional accounts emphasize differences across phonological, motor-based, and articulation profiles [2,6,67]. Intervention focus and intensity may also vary by clinical profile and treatment approach [14,15]. Treatment evidence for CAS suggests that some children may require more intensive or specialized clinician-guided intervention [68]. This study identified shared collaborative processes across participants but did not compare these processes by diagnostic subtype or treatment approach.

Family and service constraints also shaped how collaboration was sustained. Studies on parent-mediated interventions have reported challenges related to competing demands on parents’ time, children’s behavior, and alignment of goals [69]. Recent work on family-centered planning similarly highlights implementation challenges for health professionals and families [30]. In this study, background constraints, including workload and burnout, emotional burden, and scheduling constraints, shaped parents’ and SLPs’ capacity to sustain therapy-related work. Practice-level constraints, including gaps in feedback continuity, limited visibility of home practice and progress, and material and documentation management burden, shaped how SLPs and parents coordinated practice across settings. These findings suggest that home practice should be introduced with attention to family routines and service constraints to avoid increasing parental burden while extending practice beyond SLP sessions.

### Implications for Digital Support

The practice-level constraints identified in this study suggest possible implications for digital support in pediatric SSD intervention. Participants described gaps in feedback continuity, limited visibility of home practice and progress, and burdens associated with material and documentation management. Human–computer interaction research involving pediatric speech-language pathology has highlighted the need to design digital tools that respond to the abilities and contexts of children with disabilities [70]. Related work on parent-clinician communication suggests that visualization tools may support communication when clinical information is difficult to share or interpret across stakeholders [71]. Digital tools could be explored as supports for feedback continuity, visibility of home practice and progress, and material and documentation management across SLP sessions and home practice. Recent work on automatic speech recognition assessment for children with SSD also suggests the relevance of digital assessment tools for speech-related monitoring and feedback [13]. Design-based research on children’s apps emphasizes iterative development and attention to child-centered use contexts [72]. Research on children’s digital technologies also suggests that adults’ roles in selecting, managing, and implementing tools should be considered during design [73]. These perspectives are relevant because digital support in pediatric SSD intervention would need to consider the practice-level constraints identified in this study, including feedback continuity across settings, visibility of home practice and progress, and material and documentation management. These possibilities should be interpreted as design implications for future research.

### Limitations

The findings should be interpreted while accounting for the limitations of the study. First, children’s perspectives were examined indirectly through parent and SLP reports due to age and speech-language limitations that constrained direct interviewing. Although this approach provided insights into children’s participation within adult–child interactions, it only partially captured children’s lived experiences and interpretations of the intervention. Future studies should incorporate developmentally appropriate and creative methods to directly elicit children’s perspectives.

Second, this study was conducted within the clinical and cultural context of South Korea; therefore, transferability to other linguistic or health care settings should be considered with caution. Although SSD is a heterogeneous clinical category, formal subtype classification was not routinely conducted within the participants’ settings, and subtype-specific differences were not examined. While the study identified common collaborative processes, coordination may vary according to clinical presentation and service context. Information on parents’ socioeconomic background was not collected, and variation in these contextual factors may also influence collaborative processes. Future research should examine how socioeconomic variation shapes collaboration across service contexts.

Third, the category integration diagram should be interpreted as a conceptual representation derived from qualitative data. The diagram clarifies relationships among the core category, action/interaction strategies, and contextual concerns identified in this study, but it does not establish feasibility, effectiveness, or implementation requirements across service contexts. Similarly, the digital support implications were inferred from practice-level constraints and participant-described examples. Future research should examine how collaborative processes and potential digital supports operate across SLP sessions and home practice, including their fit with family routines, service delivery constraints, and digital implementation conditions.

### Conclusions

This study identified striving for triadic collaboration as the central phenomenon through which SLPs and parents coordinated roles, expectations, and practice while supporting children’s participation across SLP sessions and home practice. Three action/interaction strategies—SLP-led monitoring strategies, parent-led home-based intervention strategies, and gradual motivation strategies—were organized around this core category and shaped by background and practice-level constraints. The category integration diagram represents how collaborative processes were organized in relation to the gap between structured target sound production and everyday speech use. These findings provide a process-oriented account of collaboration in pediatric SSD intervention and highlight the relevance of coordinated SLP–parent collaboration and children’s participation for supporting practice across settings in relation to speech sound generalization.

## Appendix

Multimedia Appendix 1 COREQ checklist.

Multimedia Appendix 2 Semistructured interview protocol for speech-language pathologists and parents.

Multimedia Appendix 3 Initial coding framework generated during open coding, comprising 47 provisional categories and 227 codes prior to iterative refinement.

Multimedia Appendix 4 Participant characteristics and contextual information for children described in parent interviews.

Multimedia Appendix 5 Paradigm model developed through axial coding, illustrating relationships among causal conditions, clinical contexts, intervening conditions, action/interaction strategies, and consequences.

## Abbreviations

CAS: childhood apraxia of speech
COREQ: Consolidated Criteria for Reporting Qualitative Research
GT: grounded theory
SLP: speech-language pathologist
SLT: speech-language therapy
SSD: speech sound disorder

## Data Availability

The qualitative datasets generated and analyzed during this study are not publicly available due to confidentiality protocols approved by the institutional review board and restrictions specified in the participant consent forms, but deidentified data are available from the corresponding author on reasonable request, subject to ethics approval and applicable data-sharing agreements. Deidentified underlying study data may also be provided to the journal for inspection or verification purposes.
