# Supplementary material for: Striving for Triadic Collaboration in Pediatric Speech Sound Disorder Intervention: Grounded Theory Study
Source: JMIR Pediatr Parent. 2026 Jul 8;9:e86364. doi: 10.2196/86364 (PMC13392537; doi:10.2196/86364)
Supplement: Multimedia Appendix 1 [file pediatrics_v9i1e86364_app1.docx]

Consolidated criteria for reporting qualitative studies (COREQ): 32-item checklist

Developed based on:

Tong A, Sainsbury P, Craig J. Consolidated criteria for reporting qualitative research (COREQ): a 32-item checklist for interviews and focus groups. International Journal for Quality in Health Care. 2007. Volume 19, Number 6: pp. 349–357.

| No item | Guide questions/description | Page # |
| --- | --- | --- |
|  |  |  |
| **Domain 1: Research team and reflexivity** |  |  |
| *Personal characteristics* |  |  |
| 1. Interviewer/facilitator | Which author/s conducted the interview or focus group? | 5 |
| 2. Credentials | What were the researcher’s credentials (eg, PhD and MD)? | 4 |
| 3. Occupation | What was their occupation at the time of the study? | 4 |
| 4. Gender | Was the researcher male or female? | N/A |
| 5. Experience and training | What experience or training did the researcher have? | 4 |
| *Relationship with participants* |  |  |
| 6. Relationship established | Was a relationship established prior to study commencement? | 5 |
| 7. Participant knowledge of the interviewer | What did the participants know about the researcher? | N/A |
| 8. Interviewer characteristics | What characteristics were reported about the interviewer/facilitator? | N/A |
| **Domain 2: Study design** |  |  |
| *Theoretical framework* |  |  |
| 9. Methodological orientation and theory | What methodological orientation was stated to underpin the study? | 4 |
| *Participant selection* |  |  |
| 10. Sampling | How were participants selected? | 4, 5 |
| 11. Method of approach | How were participants approached? | 4 |
| 12. Sample size | How many participants were in the study? | 4 |
| 13. Non-participation | How many people refused to participate or dropped out? Reasons? | N/A |
| *Setting* |  |  |
| 14. Setting of data collection | Where were the data collected? | 5 |
| 15. Presence of non-participants | Was anyone else present besides the participants and researchers? | N/A |
| 16. Description of sample | What are the important characteristics of the sample? | 6, 7  Multimedia Appendix 3 |
| *Data collection* |  |  |
| 17. Interview guide | Were questions, prompts, or guides provided by the authors? Were they pilot tested? | 5  Multimedia Appendix 1 |
| 18. Repeat interviews | Were repeat interviews carried out? If yes, how many? | 5 |
| 19. Audio/visual recording | Did the research use audio or visual recording to collect the data? | 5 |
| 20. Field notes | Were field notes made during and/or after the interview or focus group? | 5 |
| 21. Duration | What was the duration of the interviews or focus group? | 5 |
| 22. Data saturation | Was data saturation discussed? | 5 |
| 23. Transcripts returned | Were transcripts returned to participants for comment and/or correction? | N/A |
| **Domain 3: Analysis and findings** |  |  |
| *Data analysis* |  |  |
| 24. Number of data coders | How many data coders coded the data? | 6 |
| 25. Description of the coding tree | Did authors provide a description of the coding tree? | 6  Multimedia Appendix 2 |
| 26. Derivation of themes | Were themes identified in advance or derived from the data? | 5 |
| 27. Software | What software, if applicable, was used to manage the data? | 6 |
| 28. Participant checking | Did participants provide feedback on the findings? | 4 |
| *Reporting* |  |  |
| 29. Quotations presented | Were participant quotations presented to illustrate the themes/findings? Was each quotation identified? | 7–18 |
| 30. Data and findings consistency | Was there consistency between the data presented and the findings? | 7–20 |
| 31. Clarity of major themes | Were major themes clearly presented in the findings? | 7–18 |
| 32. Clarity of minor themes | Is there a description of diverse cases or discussion of minor themes? | 7–18 |
