# Supplementary material for: Striving for Triadic Collaboration in Pediatric Speech Sound Disorder Intervention: Grounded Theory Study
Source: JMIR Pediatr Parent. 2026 Jul 8;9:e86364. doi: 10.2196/86364 (PMC13392537; doi:10.2196/86364)
Supplement: Multimedia Appendix 2 [file pediatrics_v9i1e86364_app2.docx]

Semistructured interview protocol for speech–language pathologists (SLPs) and parents.

This appendix presents the initial interview guide used to support early data collection. Consistent with grounded theory methodology, questions were iteratively refined during data collection and analysis. Prequestionnaire responses informed the tailoring of interview questions to participants’ experiences and contexts.

### 1. Interview questions for SLPs.

| Domain | Interview guideline | Probe questions |
| --- | --- | --- |
| **Opening questions** |  |  |
|  | - Could you describe the typical profiles of children you provide therapy for? | - What kinds of speech or language difficulties or diagnoses do you most frequently encounter in clinical practice? - What are the typical age ranges and genders of the children you usually provide therapy for? |
| **Domain 1. How do parents and SLPs differ in their perceptions of the speech therapy experience?** |  |  |
|  | - Can you describe how speech therapy for children with speech sound disorders is typically conducted in your practice? | - Could you walk me through how you typically assess children with speech sound disorders? - How do you usually structure and deliver therapy sessions for these children? |
|  | - What factors do you think most influence the outcomes of speech therapy for children with SSDs? | - What child-related factors tend to influence therapy outcomes? - How does parental involvement affect a child’s progress in therapy? |
| **Domain 2. What factors hinder or support collaboration between parents and SLPs?** |  |  |
|  | - How do you typically involve parents in the therapy process? | - What types of information or guidance do you usually provide to parents? - Do you adjust your communication approach according to the parent’s level of engagement or understanding? - Have you ever had to navigate conflicting expectations with a parent? How did you handle it? |
|  | - Can you share any memorable experiences—successful or challenging—when working with parents? | - What made this collaboration work well (or not)? - How did you address difficulties or misunderstandings? |
| **Domain 3. How are therapy and practice activities conducted in clinics and home settings?** |  |  |
|  | - What approaches have you found most effective in delivering therapy during sessions? | - Please mention any specific techniques or materials that you frequently use. - How do you adjust your approach based on the child’s characteristics or responses? - How do you typically explain the therapy process and outcomes to parents? |
|  | - What types of home practice activities do you recommend to parents? | - What types of materials or activities do you usually suggest? - How frequently do you typically recommend parents to conduct home practice with their child? - What challenges do parents commonly face when trying to implement home practice? |
|  | - How do you monitor or follow up on children’s home practice activities? | - How do you typically receive updates or feedback from parents about home practice? - How do you use this information in therapy planning? |
| **Domain 4. How do parents and SLPs use digital tools in therapy?** |  |  |
|  | - Have you used digital devices or content (eg, apps, videos, and AI tools) during therapy sessions or as part of home practice? | - What are your reasons for using or not using digital tools? - What types of digital tools do you typically use? - How do children usually respond to the use of digital tools? - What factors do you think influence children’s positive or negative reactions to these tools? |
|  | - What are the advantages or disadvantages of using digital tools in speech therapy? | - How do digital tools support therapy for children with speech sound disorders? - How do parents generally respond to the use of digital tools? - Have you experienced any limitations or challenges when using these tools? - If you could design a speech practice app yourself, what features would you want to include? |
| **Domain 5. How is child engagement promoted across clinics and home settings?** |  |  |
|  | - What strategies do you use to help children stay motivated and actively engaged during therapy sessions? | - What have you found effective in maintaining a child’s motivation in therapy? - What factors tend to make staying focused or engaged difficult for children during sessions? |
|  | - How do you support families in maintaining a child’s motivation for speech practice at home? | - What approaches or suggestions do you usually provide to help with home practice? - How do you help parents when their child resists or refuses to practice at home? |

### 2. Interview questions for parents

| Domain | Interview guideline | Probing questions |
| --- | --- | --- |
| **Opening questions** |  |  |
|  | - Can you describe how your child’s speech and language developed over time? | - How did you first notice that your child may be having difficulty with pronunciation? - Can you describe a situation in which your child’s pronunciation caused challenges in communication or daily life? |
| **Domain 1. How do parents and SLPs differ in their perceptions of the speech therapy experience?** |  |  |
|  | - Can you tell me about your child’s overall experience with professional speech therapy? | - How did your expectations or feelings change over the course of therapy? - Can you share any particularly impressive or memorable moments centered on your child’s changes? - Can you describe a moment that made you feel especially happy or frustrated during your child’s speech therapy? |
|  | - Can you tell me about any aspects of the therapy process that you found helpful or challenging? | - What parts of the therapy did you feel worked or did not work well for your child? - What types of support during therapy felt most helpful to you, and did you encounter challenges or frustrations? |
| **Domain 2. What factors hinder or support collaboration between parents and SLPs?** |  |  |
|  | - How do you communicate with your child’s SLP about your child’s condition and progress? | - How has communication with the SLP helped you understand your child’s progress? - Were there moments when you and the SLP did not seem to be on the same page? What happened? - Can you describe an experience that strengthened or weakened your trust in the SLP? |
|  | - Can you tell me what it was like to work together with your child’s SLP during the therapy process? | - What helped you feel more confident during the therapy journey? - Were there moments that made you feel especially challenged or concerned? |
| **Domain 3. How are therapy and practice activities conducted in clinics and home settings?** |  |  |
|  | - Can you describe how your child’s speech therapy sessions are typically conducted at the clinic? | - How is information about your child's therapy sessions shared with you? - Does your child’s SLP assign any home practice tasks for you to work on with your child? - What types of materials or resources does the SLP provide to support home practice? |
|  | - What home activities promote your child’s speech development? | - Who usually participates in your child’s speech practice at home, and how do you support their development? - What types of activities have been most helpful for your child’s speech practice at home? - How do you fit speech practice into your daily routine? |
| **Domain 4. How do parents and SLPs use digital tools in therapy?** |  |  |
|  | - Have you used digital devices or contents (eg, apps, videos, and AI tools) for speech practice? | - What made you choose the digital tools you used for your child’s speech practice? - Did the tools meet your expectations? Why or why not? - How did your child respond to using the digital tools? - What do you think influenced your child’s positive or negative reaction? |
|  | - What has been your experience using these digital tools? | - What aspects of using digital tools for speech practice did you find particularly helpful? - Were there any aspects of using digital tools for speech practice that you found difficult or ineffective? - If you could design a speech practice app, what features or functions would you like to be included? |
| **Domain 5. How is child engagement promoted across clinics and home settings?** |  |  |
|  | - How do you help your child stay motivated to attend therapy sessions consistently? | - What helped your child stay motivated for therapy? - What makes staying engaged in therapy difficult for your child? |
|  | - How do you maintain your child’s motivation for speech practice at home? | - What approaches have you found helpful for encouraging home practice? - How do you handle times when your child resists practicing at home? |
