# Supplementary material for: Striving for Triadic Collaboration in Pediatric Speech Sound Disorder Intervention: Grounded Theory Study
Source: JMIR Pediatr Parent. 2026 Jul 8;9:e86364. doi: 10.2196/86364 (PMC13392537; doi:10.2196/86364)
Supplement: Multimedia Appendix 3 [file pediatrics_v9i1e86364_app3.docx]

This appendix presents the initial coding framework generated during open coding. It includes 47 provisional categories and 227 codes, which were refined through constant comparison and analytic discussions.

| No. | Provisional Categories | Codes |
| --- | --- | --- |
| 1 | Initiation of therapy | 1.1 Recommendation from others (eg, parents and preschool teachers)  1.2 Comparison with peers  1.3 Peer rejection or isolation  1.4 Decline in self-esteem due to repeated failure in interaction  1.5 Behavioral problems due to repeated failure in interaction (self-centeredness, aggression, and irritability)  1.6 Underestimation of abilities due to issues in speech articulation  1.7 Initiation after infant health screening → recommendation for formal assessment → start of therapy |
| 2 | Speech acquisition environment | 2.1 Presence or absence of siblings  2.2 Financial circumstances  2.3 Parents’ knowledge of speech–language therapy  2.4 Parenting style, values, and attitudes  2.5 Parents’ subjective overestimation of the child’s abilities  2.6 Parents’ high comprehension of the child’s speech |
| 3 | Child characteristics at therapy initiation | 3.1 Chronological age and gender  3.2 Level of literacy (Korean alphabet acquisition)  3.3 Social interaction skills  3.4 Speech symptoms/severity/causes  3.5 Child’s temperament and traits (eg, sensitivity and stubbornness)  3.6 Comparison with peers  3.7 Presence of co-occurring disabilities  3.8 Presence of language delay  3.9 Self-monitoring abilities  3.10 Levels of attention and concentration  3.11 Cognitive levels  3.12 Child’s acceptance of therapy |
| 4 | Parental guilt | 4.1 Lack of professional knowledge for providing guidance (place and manner of articulation)  4.2 Insufficient performance in home activities  4.3 Difficulty in maintaining initial enthusiasm and regulating emotions  4.4 Delay in starting therapy  4.5 Lack of patience (difficulty in waiting for progress) |
| 5 | Acquisition of professional knowledge and experience by parents | 5.1 Desire to provide the best possible support  5.2 Reality in which therapy practice becomes prioritized over child-led interaction  5.3 Frustration due to difficulty in accessing information in specialized fields |
| 6 | Speech therapy environment | 6.1 Parents’ ability to collect educational resources  6.2 Lower priority compared with academic schooling  6.3 Child’s busy schedule interfering with therapy |
| 7 | Reasons for choosing a therapy center | 7.1 Satisfaction with the therapy environment (eg, space and facilities)  7.2 Satisfaction with the SLP (eg, knowledge, attitude, and personality)  7.3 Satisfaction with therapy costs  7.4 Recommendations or reviews from others |
| 8 | Parental challenges in initial therapy decisions | 8.1 Issues related to waitlists, costs, distance, and scheduling  8.2 Managing concurrent therapies (eg, play, cognitive, occupational, and developmental therapies and art/music/sports activities)  8.3 Difficulty in assessing SLPs’ styles (directive vs. interactive) and expertise  8.4 Challenges in selecting appropriate centers and SLPs |
| 9 | Discrepancies between expectations and reality | 9.1 No provision of full formal test results  9.2 Superficial feedback immediately after assessment  9.3 Free diagnostic assessment services offered  9.4 Frustration due to lack of verifiable evidence of therapy effectiveness relative to costs |
| 10 | Initial assessment | 10.1 Types of information required for accurate diagnosis (eg, speech sound recordings and videos of mouth movements)  10.2 Level of responsiveness to stimulation  10.3 Error patterns (eg, developmental, non-developmental, or mixed)  10.4 Child’s speech intelligibility |
| 11 | Initial goal setting (level-specific and stage-specific therapy planning) | 11.1 Objective assessment of the child’s articulation level (required for parents and SLPs)  11.2 Therapy planning that considers the individual characteristics of children  11.3 Insufficient diagnostic testing for co-occurring disabilities  11.4 Challenges in identifying the causes of articulation errors  11.5 Lack of systematic curriculum for therapy  11.6 Targeted goal for the completion of therapy prior to school entry  11.7 Hierarchical articulation therapy planning (sentence-level practice including target sounds)  11.8 Automated goal-setting tools (support systems for SLPs)  11.9 Greater emphasis on language delay over articulation errors  11.10 Adjustment of therapy frequency and intensity (with a consideration of therapy duration and child’s attention span) |
| 12 | Personalization of therapy | 12.1 Diverse cases of errors that require individualized approaches  12.2 Adjustments in therapy based on child-specific traits  12.3 Time required for preparing customized materials for each child |
| 13 | Instructional materials supporting therapy | 13.1 Use of picture cards, story cards, tongue depressors, books, board games, and toys  13.2 Timely provision of cues and prompts during therapy  13.3 Support services for material creation (eg, Do Speech)  13.4 Provision of diverse speech sound models (by gender, age, and regional accents)  13.5 Modeling in various real-life contexts and locations  13.6 Use of *error notebooks* for tracking misarticulation  13.7 Use of mirrors, video recording, photo capturing, Snow app filters, Google voice search, and Galaxy Note in differentiating correct/incorrect articulation  13.8 Utilizing resources such as Google search and design tools (eg, Mirae Canvas)  13.9 Sharing of resources between therapists and parents |
| 14 | Utilization of online services | 14.1 Appropriateness of digital learning content  14.2 Need for control over device use (eg, operations, screen time, and content type)  14.3 Automation of time management  14.4 Automation of assignment delivery  14.5 Reduction of variability in SLP experiences through standardized digital support |
| 15 | Acceptance of digital services | 15.1 Low acceptance among SLPs  15.2 High acceptance among SLPs  15.3 Low acceptance among parents  15.4 High acceptance among parents |
| 16 | Building rapport and empathy | 16.1 Rapport between SLPs and children, between SLPs and parents, and between parents and children  16.2 Triggers for establishing mutual rapport  16.3 Factors that influence rapport development |
| 17 | Parental counseling | 17.1 Difficulty in understanding professional terminologies  17.2 Lack of sufficient information for parents (eg, evaluation results, progress updates, and session contents)  17.3 Materials that enable parents to intuitively comprehend the child’s condition  17.4 Communication of session contents to parents  17.5 Detailed explanations of the articulation status of the child  17.6 Consideration of the child’s emotional state during joint counseling sessions  17.7 Insufficient counseling time  17.8 Lack of task feedback due to the busy schedules of SLPs |
| 18 | Goal agreement | 18.1 Goal setting among SLPs, parents, and children  18.2 Agreement on sessional, short-term, long-term, and final discharge goals  18.3 Disagreement on the timing of therapy discharge  18.4 Final agreement on therapy objectives (successful social communication) |
| 19 | Parental education | 19.1 Teaching parents the methods for articulation guidance  19.2 Coaching parents on providing feedback according to various situations  19.3 Explaining articulation points and methods, including modeling techniques  19.4 Techniques for providing corrective feedback on misarticulation |
| 20 | Therapy content and task instruction | 20.1 Discrimination training, imitation elicitation, breath/phonation control guidance  20.2 Providing auditory, visual, and articulatory prompts  20.3 Corrective feedback on articulation errors (accurate/inaccurate articulations and responses)  20.4 Provision of customized homework assignments  20.5 Instruction on slower speech rate control  20.6 Implementation of oral–motor exercises  20.7 Encouragement of self-monitoring of speech sounds  20.8 Strengthening memory through repetitive practice  20.9 Management of problem behaviors and practice of emotional regulation  20.10 Provision of accurate speech models  20.11 Monitoring learned articulation errors  20.12 Discovery of additional misarticulation through continuous observation  20.13 Identification of causes and habits that contribute to articulation problems |
| 21 | Factors influencing concentration | 21.1 Learning environment/tools (therapy room versus home, device use, and presence of SLP)  21.2 Intrinsic motivation (eg, motivation to finish quickly and play)  21.3 Differences in attention span according to age |
| 22 | Enhancing concentration through interest induction | 22.1 Gamification, AI interaction, tablet use, battles, activities, play, sticker rewards, stamps  22.2 Utilizing children’s preferred toys/games (eg, Pokémon and TiniPing)  22.3 Provision of optimal challenge level (stimulation of a sense of achievement and competitiveness)  22.4 Gradual increases in task difficulty  22.5 Incorporation of elements of fun  22.6 Achievement of repetitive practice through natural participation |
| 23 | Factors leading to decreased concentration | 23.1 Simple, repetitive activities that lead to boredom  23.2 Diminished reinforcement effects due to habituation to rewards |
| 24 | Building trust | 24.1 Trust relationships between SLPs and children, between SLPs and parents, and between parents and children  24.2 Triggers for developing mutual trust  24.3 Factors that influence trust (eg, professionalism and perceived expertise) |
| 25 | Parental perceptions of SLP professionalism | 25.1 Analytical skills (identifying causes of speech issues)  25.2 Instructional leadership (child-centered approach and consideration of child's emotional state)  25.3 Application of theoretical knowledge to practice  25.4 Diversity of therapy programs customized to the individual traits of children  25.5 Ability to adjust task difficulty appropriately  25.6 Flexible responsiveness to child needs  25.7 Patience until the child is ready to engage  25.8 Deliberately planned individualized therapy  25.9 Academic background, certification level, and clinical experience  25.10 Age and personal parenting experience |
| 26 | SLP responsibility and burden | 26.1 Inadequate delivery of prepared therapy sessions  26.2 Difficulty in maintaining initial enthusiasm and emotional regulation  26.3 Anxiety regarding the timing of therapy discharge |
| 27 | Expertise in articulation therapy | 27.1 Comprehension and acquisition of articulation knowledge (place and manner of articulation)  27.2 Analysis and interpretation of evaluation results  27.3 Securing auditory sensitivity for accurate feedback  27.4 Development of customized content for individual needs  27.5 Communication and experience-sharing through small professional networks  27.6 Collaborative growth through open communities |
| 28 | Parental motivation | 28.1 Sense of pride experienced by parents  28.2 Sense of accomplishment experienced by parents |
| 29 | SLP motivation | 29.1 Sense of pride experienced by SLPs  29.2 Sense of accomplishment experienced by SLPs |
| 30 | Promoting child motivation | 30.1 Commitment to work hard with the child  30.2 Praise and encouragement for correct pronunciation and effort  30.3 Rewards (eg, free time, games, and videos) given after achieving practice goals; adjustment of the balance/intensity of practice and rewards  30.4 Permission for children to make choices (giving autonomy)  30.5 Stimulation of competitiveness  30.6 Utilization of reinforcement tools preferred by the child |
| 31 | Decrease in child motivation | 31.1 Discouragement and frustration due to correction and negative feedback |
| 32 | Progress monitoring | 32.1 Monitoring of progress (speed, attainment, and generalization)  32.2 Failure to consolidate skills due to increased phonological or syntactic complexity  32.3 Informal evaluations and observations during sessions  32.4 Formal evaluations every six months  32.5 Peer comparisons  32.6 Need for intuitive data visualization (for counseling support and rapid child assessment) |
| 33 | Goal resetting | 33.1 Goal resetting based on accurate assessment of current progress  33.2 Adjusting task difficulty levels incrementally  33.3 Flexible goal adjustments depending on circumstances |
| 34 | Parental challenges during speech therapy | 34.1 Lack of interim feedback on child’s progress  34.2 Gap between assigned tasks and actual execution at home (mismatch between the expectations of parents and SLPs)  34.3 Anxiety regarding the timing and criteria for therapy discharge |
| 35 | Child challenges during speech therapy | 35.1 Boredom due to repetitive practice |
| 36 | Active participation of the child in speech therapy | 36.1 Active participation in repetitive practice  36.2 Self-initiated completion of tasks  36.3 Anticipation and eagerness for therapy sessions |
| 37 | Home-based practice (child-focused) | 37.1 Repetition of therapy activities at home  37.2 Review and reinforcement of previous sessions  37.3 Requirements for promoting generalization  37.4 Need for consistent reminders  37.5 Opportunities for peer interaction  37.6 Acceleration of therapy outcomes through home tasks (progress and generalization)  37.7 Language stimulation through reading  37.8 Predominantly one-way communication instead of interactive exchanges at home (due to time constraints)  37.9 Challenges in completing home tasks (eg, child’s refusal, forgetting assignments, losing materials, lack of understanding of instructions, parents’ difficulty in providing feedback)  37.10 Need for communication aids to support homework completion  37.11 Regulation of task intensity (eg, frequency and duration)  37.12 Management of stress related to home practice |
| 38 | Home-based monitoring of learning (outside therapy sessions) | 38.1 Monitoring of previously learned error sounds  38.2 Discovering additional articulation errors through ongoing observation  38.3 Identifying articulation habits and underlying causes of errors  38.4 Difficulty in checking homework progress (eg, lack of verification methods, time constraints, or misleading reports from children) |
| 39 | Supporting self-monitoring | 39.1 Recognition/non-recognition of one’s articulation errors  39.2 Difficulty in speech sound discrimination and need for discrimination training  39.3 Practice in distinguishing between correct and incorrect articulation  39.4 Practice in monitoring one’s speech sounds  39.5 Consideration of the child’s cognitive level when supporting self-monitoring  39.6 Immediate feedback using familiar tools (eg, voice search)  39.7 Providing auditory cues using familiar environmental sounds  39.8 Offering visual, auditory, or behavioral cues to support self-correction  39.9 Feedback on home practice or through SLP evaluation (eg, accuracy and consistency) |
| 40 | Verifying self-monitoring ability | 40.1 Child’s ability to regulate speech rate independently  40.2 Self-correction of articulation errors  40.3 Avoidance behaviors in response to corrective feedback |
| 41 | Factors facilitating generalization | 41.1 Importance of home-based practice (critical role of parents)  41.2 Frequent practice of commonly used everyday expressions  41.3 Providing diverse speech models (eg, photos, videos, movies, and cartoons)  41.4 Emphasis on the importance of repeated practice |
| 42 | Challenges in generalization | 42.1 Difficulty in generalizing across contexts (eg, locations and conversation partners)  42.2 Difficulty in maintaining correct articulation outside the therapy room (due to lack of motivation, forgetting, and incomplete skill acquisition)  42.3 Difficulty in improving self-monitoring abilities |
| 43 | Communication intentions | 43.1 Discouragement due to negative feedback  43.2 Motivation through praise (for attempts, accuracy, and effort)  43.3 Sense of accomplishment when achieving difficult tasks  43.4 Avoidance of speech production  43.5 Frustration despite effort due to the lack of results  43.6 Substitution of gestures for verbal communication |
| 44 | Factors contributing to satisfaction with therapy outcomes | 44.1 Perception of improvement in speech abilities  44.2 Development and enhancement of self-confidence  44.3 Improvement in peer interactions  44.4 Proactive initiation of communication  44.5 Achievement of goals within the expected time frame |
| 45 | Factors facilitating goal achievement | 45.1 Improvement in articulation skills  45.2 Consistency and sustained effort |
| 46 | Factors hindering goal achievement | 46.1 Child’s physical or emotional condition  46.2 Difficulty in task execution  46.3 Child’s attention and concentration issues |
| 47 | Follow-up management after therapy discharge | 47.1 Post-discharge monitoring and management |
