# Supplementary material for: Striving for Triadic Collaboration in Pediatric Speech Sound Disorder Intervention: Grounded Theory Study
Source: JMIR Pediatr Parent. 2026 Jul 8;9:e86364. doi: 10.2196/86364 (PMC13392537; doi:10.2196/86364)
Supplement: Multimedia Appendix 4 [file pediatrics_v9i1e86364_app4.docx]

This appendix presents detailed participant characteristics and contextual information for children described in parent interviews.

### 1. Characteristics of parents (n = 10).

| Characteristics | N (%) |
| --- | --- |
| **Parent’s age group** |  |
| 30s | 7 (70) |
| 40s | 3 (30) |
| **Location** |  |
| Seoul | 6 (60) |
| Metropolitan area | 4 (40) |
| **Education level** |  |
| High school | 1 (10) |
| University degree | 6 (60) |
| Graduate education | 3 (30) |
| **Work status** |  |
| Graduate student | 1 (10) |
| Employed | 2 (20) |
| Speech–language pathologist | 2 (20) |
| Homemaker | 5 (50) |

### 2. Characteristics of SLPs^a^ (n = 12).

| Characteristics | N (%) |
| --- | --- |
| **Age group** |  |
| 20s | 8 (67) |
| 30s | 2 (17) |
| 40s | 2 (17) |
| **Location** |  |
| Seoul | 4 (33) |
| Metropolitan area | 5 (42) |
| Other | 3 (25) |
| **Education** |  |
| Bachelor’s degree or less | 3 (25) |
| Master’s degree in progress | 2 (17) |
| Master’s degree or higher | 7 (58) |
| **Current workplace** |  |
| Private clinic | 7 (58) |
| Hospital-affiliated center | 5 (42) |
| **Years of clinical experience** |  |
| Less than 4 years | 4 (33) |
| 4–8 years | 5 (42) |
| More than 8 years | 3 (25) |
| **SSD^b^ cases treated** |  |
| Less than 10 children | 2 (17) |
| 10–20 children | 4 (33) |
| More than 20 children | 6 (50) |

^a^SLP: Speech–language pathologist

^b^SSD: Speech sound disorders

### 3. Characteristics of children described in parent interviews (n=10).

| Child ID | Gender | Age | Treatment  duration | Service  setting | Mode of  delivery |
| --- | --- | --- | --- | --- | --- |
| C01 | Male | 5;4 | 4 months | Hospital-based | In-person |
| C02 | Male | 6;5 | 3 years 9 months | Private clinic | In-person |
| C03 | Male | 4;0 | 2 years | Hospital-based & Private clinic | In-person |
| C04 | Female | 4;9 | 2 years 3 months | Private clinic | In-person |
| C05^c^ | Female | 6;6 | 8 months | Home-based | Remote |
| C06 | Female | 6;4 | 2 months | Hospital-based | In-person |
| C07 | Male | 4;11 | 2 years 5 months | Private clinic | In-person |
| C08 | Male | 4;1 | 2 years | Hospital-based | In-person |
| C09 | Female | 4;0 | 1 year 7 months | Hospital-based | In-person |
| C10^c^ | Female | 5;8 | 2 years | Private clinic | In-person |

| Child ID | ICS^a^ | PCC^b^ |
| --- | --- | --- |
| C01 | 3.14 | 70.83% |
| C02 | 2.29 | 90.69% |
| C03 | 4.00 | 74.30% |
| C04 | 2.86 |  |
| C05^c^ | 4.14 |  |
| C06 | 4.29 | 90.69% |
| C07 | 4.00 |  |
| C08 | 3.14 | 71.40% |
| C09 | 3.14 |  |
| C10^c^ | 3.86 |  |

^a^ICS: Intelligibility in context scale (1–5)

^b^PCC: Percentage of consonants correct
