# Supplementary material for: Striving for Triadic Collaboration in Pediatric Speech Sound Disorder Intervention: Grounded Theory Study
Source: JMIR Pediatr Parent. 2026 Jul 8;9:e86364. doi: 10.2196/86364 (PMC13392537; doi:10.2196/86364)
Supplement: Multimedia Appendix 5 [file pediatrics_v9i1e86364_app5.docx]

This appendix presents the paradigm model developed through axial coding to explain relationships among causal conditions, clinical contexts, intervening conditions, action/interaction strategies, and consequences. It illustrates how striving for triadic collaboration is situated within causal and contextual conditions, how action/interaction strategies are enacted under intervening conditions, and how these processes are linked to child-related consequences.

### Paradigm Model of Triadic Collaboration.

| Component | Key categories | Subcategories |
| --- | --- | --- |
| **Causal conditions** |  |  |
|  | - Limitations in face-to-face therapy | - Temporal constraints of sessions - Lack of home-based observation - Insufficient session frequency |
|  | - Interactional discrepancies | - Misalignment of goal perceptions - Asymmetric communication - Ambiguity in feedback |
|  | - Challenges in speech sound generalization | - Inconsistent performance in daily contexts - Limited self-correction - Practice discontinuity |
| **Clinical context** |  |  |
|  | - Fragmented consultation time | - Brief and task-focused consultations - Limited opportunity for extended dialogue |
|  | - Cultural reliance on professional expertise | - Deference to clinician authority - Parent role positioned as implementer |
|  | - Digital ambivalence | - Perceived usefulness of digital tools - Concerns regarding overreliance on technology |
| **Central phenomenon** |  |  |
|  | - Striving for triadic collaboration | - Bridging the clinic–home gap - Formation of cooperative partnerships |
| **Intervening conditions** |  |  |
|  | - Background constraints | - Workload and burnout - Emotional burden - Scheduling constraints |
|  | - Practice-level constraints | - Gaps in feedback continuity - Limited visibility of home practice and progress - Material and documentation management burden |
| **Action/Interaction strategies** |  |  |
|  | - SLP-led monitoring strategies | - Collaborative goal setting - Transparent communication - Progress monitoring |
|  | - Parent-led home-based intervention strategies | - Parent coaching supported by SLP - Parent-led speech practice - Guided self-monitoring - Contextualized practice |
|  | - Gradual motivation strategies | - Choice-supportive interaction - Incentive-based participation - Socially mediated reinforcement |
| **Consequences** |  |  |
|  | - Treatment fidelity and continuity | - Consistency in practice - Increased home–clinic connection - Establishment of therapeutic routines |
|  | - Child as active agent | - Autonomous self-monitoring - Perceived self-efficacy - Intrinsic motivation for self-regulation |
|  | - Strengthened triadic alliance | - Trust and partnership - Shared goal alignment - Positive feedback loop |
|  | - Enhanced speech sound outcomes | - Contextual transfer of speech skills - Improved Intelligibility - Functional social communication |
